# Cold Atmospheric Plasma Apoptotic and Oxidative Effects on MCF7 and HCC1806 Human Breast Cancer Cells

**DOI:** 10.3390/ijms23031698

**Published:** 2022-02-01

**Authors:** Catarina Almeida-Ferreira, Rafael Silva-Teixeira, Ana Cristina Gonçalves, Carlos Miguel Marto, Ana Bela Sarmento-Ribeiro, Francisco Caramelo, Maria Filomena Botelho, Mafalda Laranjo

**Affiliations:** 1 Faculty of Medicine, Institute of Biophysics, University of Coimbra, 3000-548 Coimbra, Portugal; catarinalmeidaferreira@gmail.com (C.A.-F.); rafaelesteixeira@gmail.com (R.S.-T.); cmiguel.marto@uc.pt (C.M.M.); fcaramelo@fmed.uc.pt (F.C.); mfbotelho@fmed.uc.pt (M.F.B.); 2 Faculty of Medicine, Institute for Clinical and Biomedical Research (iCBR), Area of Environment, Genetics and Oncobiology (CIMAGO), University of Coimbra, 3000-548 Coimbra, Portugal; acc.goncalves@gmail.com (A.C.G.); absarmento@fmed.uc.pt (A.B.S.-R.); 3 Department of Cardiology, Hospital Center of Vila Nova de Gaia/Espinho, EPE, 4434-502 Vila Nova de Gaia, Portugal; 4 Center for Innovative Biomedicine and Biotechnology (CIBB), University of Coimbra, 3000-548 Coimbra, Portugal; 5 Clinical and Academic Centre of Coimbra (CACC), 3004-561 Coimbra, Portugal; 6 Laboratory of Oncobiology and Hematology, Faculty of Medicine, University of Coimbra, 3000-548 Coimbra, Portugal; 7 Institute of Experimental Pathology, Faculty of Medicine, University of Coimbra, 3000-548 Coimbra, Portugal

**Keywords:** breast neoplasms, cold atmospheric plasma, plasma gases, reactive nitrogen species, reactive oxygen species

## Abstract

Breast cancer (BC) is a malignant neoplasia with the highest incidence and mortality rates in women worldwide. Currently, therapies include surgery, radiotherapy, and chemotherapy, including targeted therapies in some cases. However, treatments are often associated with serious adverse effects. Looking for new options in BC treatment, we evaluated the therapeutic potential of cold atmospheric plasma (CAP) in two cell lines (MCF7 and HCC1806) with distinct histological features. Apoptosis seemed to be the most prevalent type of death, as corroborated by several biochemical features, including phosphatidylserine exposure, the disruption of mitochondrial membrane potential, an increase in BAX/BCL2 ratio and procaspase 3 loss. Moreover, the accumulation of cells in the G2/M phase of the cell cycle points to the loss of replication ability and decreased survival. Despite reported toxic concentrations of peroxides in culture media exposed to plasma, intracellular peroxide concentration was overall decreased accompanying a reduction in GSH levels shortly after plasma exposure in both cell lines. In HCC1806, elevated nitric oxide (NO) concentration accompanied by reduced superoxide levels suggests that these cells are capable of converting plasma-derived nitrites into NO that competes with superoxide dismutase (SOD) for superoxide to form peroxinitrite. The concomitant inhibition of the antioxidative activity of cells during CAP treatment, particularly the inhibition of cytochrome c oxidase with sodium azide, synergistically increased plasma toxicity. Thus, this in vitro research enlightens the therapeutic potential of CAP in the treatment of breast cancer, elucidating its possible mechanisms of action.

## 1. Introduction

Breast cancer (BC) is a heterogenous group of diseases with a high incidence rate worldwide, with 2.261.419 estimated new cases and 684.996 deaths in 2020, mostly women [1]. Intrinsic subtypes such as hormone-receptor-dependent (progesterone receptor (PR) and oestrogen receptor (ER)) expression), human epidermal growth factor receptor 2 (HER2) positive, and triple negative breast cancer (TNBC), which is PR, ER and HER2 negative, are clinically relevant because their therapeutic stratification depends on molecular diagnosis [2]. Surgery, in the setting of early BC, chemotherapy, endocrine and anti-HER2 therapy, and sometimes a combination of these, are the current therapeutic approaches [3]. Hormone-receptor-positive (HR+) is the most favourable diagnosis (stands out for its favourable prognosis), however, there is a prolonged significant risk of recurrence [4]. Endocrine therapy is established for these cases and the two most important drug categories in postmenopausal patients are tamoxifen and aromatase inhibitors [5]. BC expressing HER2 comprises about 15% of all cases [6], and treatment requires anti-HER2 monoclonal antibodies, namely, trastuzumab, sometimes associated with pertuzumab, as adjuvant or neoadjuvant therapy. More recently, lapatinib, a dual EGFR/HER2 reversible tyrosine kinase inhibitor, has been introduced as a potential option for adjuvant therapy. Chemotherapy plus trastuzumab combination and dual-targeted therapy with trastuzumab plus lapatinib in patients with locally advanced HER2-positive breast cancer shows increased complete pathologic response [7]. Nevertheless, TNBC affects 15–20% of patients, and it is associated with a worse prognosis because targeted treatment is not available, and chemotherapy is the only option [8]. Poly-ADP-ribose polymerase (PARP) inhibitors are best known as a targeted treatment for BRCA1 and BRCA2 genes, and they are in clinical trials combined with chemotherapy [3,9]. All these therapies are accompanied by unwanted side effects [10]. Thus, the research on new therapies is crucial, and recently, cold plasma has emerged as a novel approach for anticancer therapy with a selective potential regarding phenotypically normal cells [11,12].

Plasma, commonly known as the fourth state of matter, has enough energy to ionize a significant amount of charged particles, being able to generate reactive oxygen and nitrogen species, and its future applications in field of biomedicine are promising [13]. There are two types of plasma: atmospheric pressure and low pressure. One of the main differences between them is the mean free paths between electrons and heavy particles that are extremely short in atmospheric pressure plasma, promoting collision among particles [14]. Within these, there are two distinct models: thermal and non-thermal plasmas. The core gas temperatures in thermal plasmas are above 10.000 K, and in non-thermal plasma, the sensible temperature remains at room temperature [14]. Moreover, plasma has a wide range of applications such as the use as surface disinfectants in healthcare facilities, even though more tests are required in order to prove its safety and efficacy [15].

Recent studies suggest an interesting potential of cold atmospheric plasma (CAP) in cancer therapy, as demonstrated by the selective eradication of cancer cells in vitro [16,17,18,19,20]. Previously, we studied the effect of cold plasma in different human cancer cell lines including BC, melanoma, colon carcinoma and prostate cancer. The results showed a significant decrease in cell viability in most cell lines. Particularly, the viability of BC cells decreased following only 90 s of CAP exposure [19]. Furthermore, a robust study involving direct and indirect CAP treatment of retinoblastoma cells and phenotypically normal counterparts suggests that this therapy has the potential to selectively ablate tumour cells [20]. Thus, the main goal of this study was to assess the biological outcome and molecular mechanisms of action of CAP in BC cell lines, namely, hormonal-receptor-positive breast cancer cell line MCF7 and triple-negative breast cancer cell line HCC1806.

## 2. Results

### 2.1. CAP Induced Apoptosis and Increased Apoptotic Factors in Breast Cancer Cells

**Figure 1:**
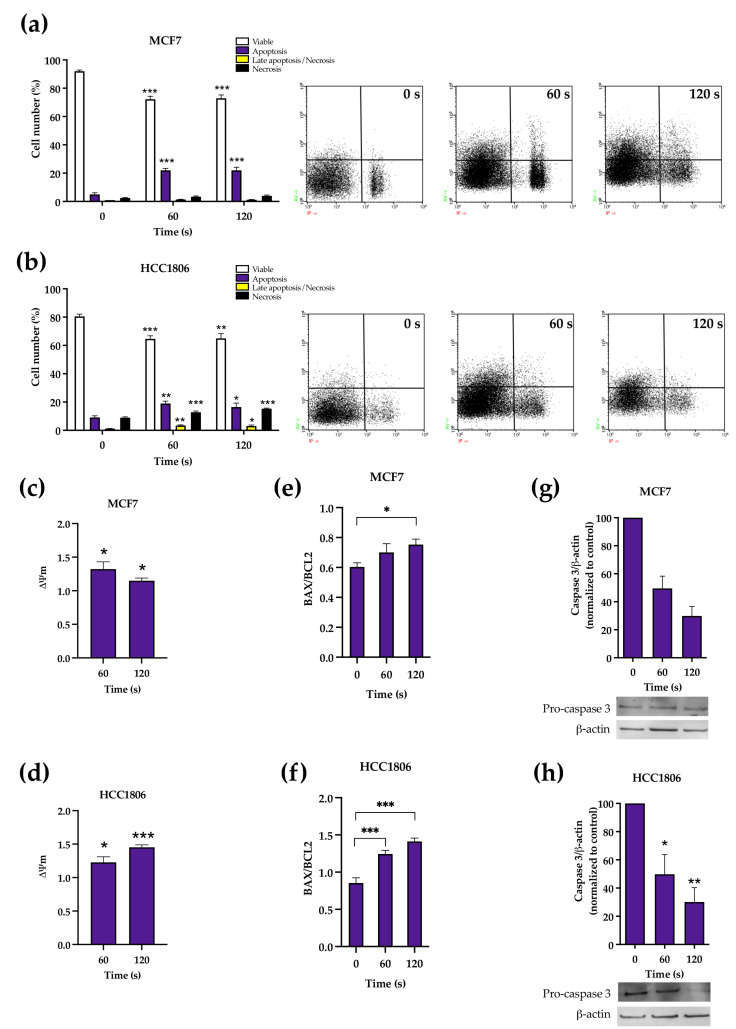
Types of cell death (**a**,**b**), mitochondrial membrane potential (**c**,**d**), BAX/BCL2 ratio (**e**,**f**) and procaspase 3 expression in MCF7 (**a**,**c**,**e**,**g**) and HCC1806 (**b**,**d**,**f**,**h**) cell lines was assessed by flow cytometry 24 h after treatment. Cell death results express the percentage of viable cells, apoptosis, late apoptosis/necrosis and necrosis of at least two independent experiments; representative dot-plots of the annexin-V and propidium iodide labelling are shown. Mitochondrial membrane potential represents the ratio between monomers and aggregates of at least three independent assays. Mitochondrial membrane potential results are presented normalized to the value 1 corresponding to control cell cultures not exposed to cold atmospheric plasma (CAP). BAX/BCL2 ratio represent at least three independent experiments. Types of cell death, mitochondrial membrane potential and BAX/BCL2 ratio were evaluated by flow cytometry. Analysis of procaspase 3 expression was performed by Western blot. Results are presented as the mean ± SE. Statistically significant differences are shown with * *p* < 0.05, ** *p* < 0.01 and *** *p* < 0.001.

CAP therapy led to a decrease in viable cells on both cell lines, as shown in Figure 1a,b. In MCF7-treated cells, this reduction in viability was followed by an increase in apoptosis, while in HCC1806-treated cells the most common type of death varied with CAP exposure time. Viable MCF7 cells decreased from 92.00 ± 0.84% to 72.25 ± 2.08% (*p* < 0.0001) after 60 s of CAP exposure and 72.88 ± 2.38% (*p* < 0.0001) after 120 s, while apoptosis increased from 5.00 ± 1.10% to 22.00 ± 1.44% (*p* = 0.39) after 60 s of exposure and 22.00 ± 2.16% (*p* = 0.34) after 120 s.

Regarding HCC1806, the proportion of viable cells significantly decreased from 80.50 ± 1.59% to 64.67 ± 2.16% (*p* = 0.0008) after 60 s of exposure and to 65.00 ± 3.39 % (*p* = 0.01) after 120 s of exposure. The treatment also induced a significant increase in cells in apoptosis after 60 s from 9.17 ± 1.11% to 19.00 ± 1.67% (*p* = 0.005), and after 120 s to 16.5 ± 2.72 (*p* = 0.03). Cell population in late apoptosis/necrosis increased significantly after 60 s of exposure from 1.17 ± 0.17% to 3.50 ± 0.34% (*p* = 0.008) and to 3.17 ± 0.60% (*p* = 0.028) after 120 s of treatment. The number of cells in necrosis increased significantly after 60 and 120 s of exposure to cold plasma, from 9.00 ± 0.52% to 12.83 ± 0.79% (*p* = 0.0008) and 15.33 ± 0.42% (*p* = 0.006).

In addition, MCF7 cells showed a significant increase in the monomers/aggregates (M/A) ratio compared to the control, 132.19 ± 10.74% (*p* = 0.0239) after 60 s of exposure to cold plasma and 114.84 ± 4.05% (*p* = 0.01) after 120 s of treatment, following the loss of mitochondrial membrane potential (MMP). Likewise, the HCC1806 cell line displayed a significant loss of MMP under the same conditions of plasma treatment. After 60 and 120 s, the M/A ratios were 122.66 ± 8.33% (*p* = 0.0156) and 145.35 ± 3.63% (*p* < 0.0001), respectively (see Figure 1c,d).

An increase in the MCF7 intracellular BAX/BCL2 ratio was observed after 120 s of exposure to cold plasma, from 60.33 ± 2.84% to 75.33 ± 3.64% (*p* = 0.048). HCC1806 intracellular BAX/BCL2 ratio increased when compared to control, from 85.43 ± 6.94% to 124.47 ± 4.91% (*p* = 0.0004) after 60 s of exposure and to 141.22 ± 4.62% (*p* < 0.0001) after 120 s of CAP treatment (see Figure 1e,f).

Pro-caspase 3 expression was decreased by more than half after 60 and 120 s of CAP exposure on both cell lines (see Figure 1g,h), which is compatible with the cleavage and activation of this effector protein. After 60 and 120 s of CAP exposure, pro-caspase 3 normalized expression reduced to 49.50 ± 8.79% (*p* = 0.11, n = 2, df = 1) and to 29.84 ± 6.75% (*p* = 0.06, n = 2, df = 1), respectively, in MCF7 cells and to 49.80 ± 13.90% (*p* = 0.0364, n = 3) and to 30.14 ± 10.13% (*p* = 0.0062, n = 3), respectively, in HCC1806 cells.

### 2.2. Plasma Treatment May Induce Different Patterns of Nitroxidative Stress in Breast Cancer Cells

#### 2.2.1. Intracellular Peroxides

**Figure 2:**
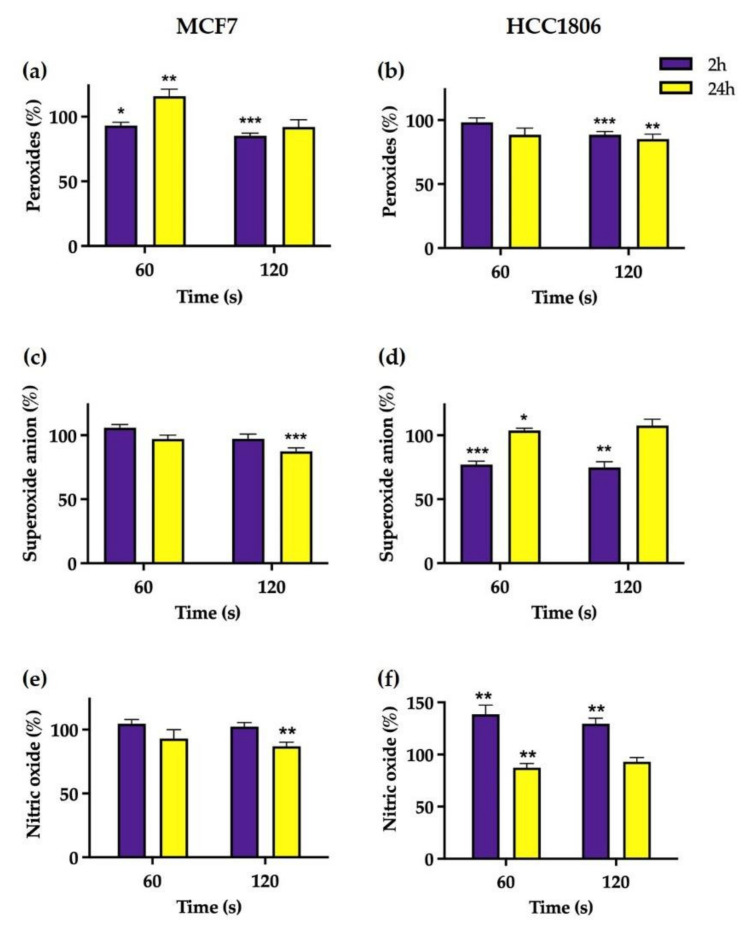
Intracellular concentration of reactive species in MCF7 (**a**,**c**,**e**) and HCC1806 (**b**,**d**,**f**) breast cancer cell lines 2 and 24 h after exposure to cold atmospheric plasma (CAP). Intracellular content of peroxide (**a**,**b**), superoxide anion (**c**,**d**) and nitric oxide (**e**,**f**) were assessed by fluorescence using DCH2-DA, DHE and DAF-FM diacetate probes, respectively. Results are presented normalized to the value 100% corresponding to control cell cultures not exposed to CAP. Data express the mean ±SE of at least two independent experiments per cell line. Statistically significant differences are shown with * *p* < 0.05, ** *p* < 0.01 and *** *p* < 0.001.

As seen in Figure 2a,b, a significant reduction in intracellular peroxides concentration of MCF7 cells was seen 2 h after treatment to 93.07 ± 2.53 % (*p* = 0.019) and 84.98 ± 2.09% (*p* < 0.0001) for both exposure periods tested and after 60 and 120 s, respectively, when compared to control. Later, after 24 h of CAP exposure, peroxide concentration normalized for MCF7 cells exposed to CAP during 120 s, while increasing for those exposed during 60 s to 115.90 ± 5.30% (*p* = 0.0049). In HCC1806 cells, a slight reduction in intracellular peroxides was observed only in cells exposed to CAP during 120 s, either 2 or 24 h after treatment to 88.54 ± 2.54% (*p* = 0.0024) and 85.17 ± 3.79% (*p* = 0.0024), respectively, when compared to the control.

#### 2.2.2. Intracellular Superoxide Anion

The intracellular superoxide concentration in MCF7-treated cells remained largely similar to the control across all the conditions, except for a slight decrease 24 h after a 120 s CAP exposition to 87.45 ± 2.61% (*p* = 0.0006), as seen in Figure 2c,d. 

An opposite pattern emerged in HCC1806 cells, with a significant decrease in the superoxide concentration 2 h after 60 s treatment to 77.17 ± 2.66% (*p* < 0.0001) and to 74.92 ± 4.84% (*p* = 0.0002) for 120 s exposure when compared to the control. After 24 h, superoxide concentration normalized in cells treated with the longest exposure while marginally increasing to 103.80 ± 1.69% (*p* = 0.045) in cells exposed to 60 s of CAP.

#### 2.2.3. Intracellular Nitric Oxide

Parallel to superoxide concentration, no significant change was seen in intracellular nitric oxide concentration of MCF7 treated cell when compared to control, except for a slight reduction to 87.00 ± 3.17% (*p* = 0.003) 24 h after 120 s of CAP exposure. However, nitric oxide concentration in HCC1806-treated cells increased to 138.79 ± 8.70% (*p* = 0.007) and 129.60 ± 5.28% (*p* = 0.001) 2 h after 60 s and 120 s exposure, respectively. Later, 24 h after treatment, the superoxide concentration decreased to 87.43 ± 3.95% (*p* = 0.0189) in HCC1806 cells treated with 60 s exposure but normalized in those cells treated with the longest exposure time.

#### 2.2.4. Inhibition of Cytochrome c Oxidase

**Figure 3:**
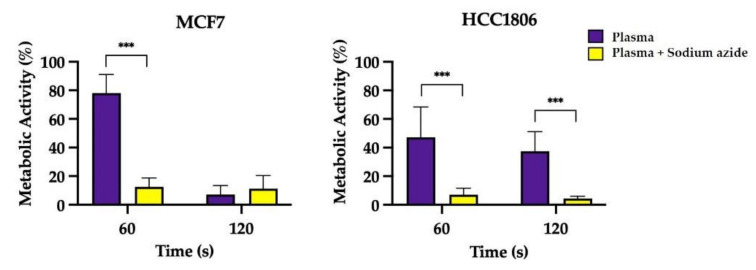
Evaluation of metabolic activity of MCF7 and HCC1806 cells following plasma treatment while incubated with sodium azide (NaN_3_), an inhibitor of cytochrome c oxidase. Results are presented normalized to the value 100% corresponding to control cell cultures not exposed to cold atmospheric plasma (CAP). Data express the mean ± SE of three independent assays. Results of cell cultures exposed to CAP in the presence of sodium azide were compared with metabolic activity of the treatment alone [19]. Statistically significant differences are shown with *** *p* < 0.001.

The results presented in Figure 3 about the incubation of cells with sodium azide during CAP treatment suggest a synergetic decrease in metabolic activity beyond that obtained with CAP exposure alone in both cell lines. When sodium azide, an inhibitor of cytochrome c oxidase, was added to cells while exposing them to 60 s of CAP treatment, metabolic activity decreased from 78.09 ± 4.12% to 14.09 ± 1.55% (*p* < 0.001) in MCF7 cells and decreased from 47.17 ± 7.53% to 7.05 ± 1.59% (*p* < 0.001) in triple-negative HCC1806 cells. A similar trend was observed in HCC1806 cells treated with a longer CAP exposure.

### 2.3. Evaluated Antioxidative Defences Did Not Change in Response to Nitroxidative Stress in the First 24 h following CAP Exposure

**Figure 4:**
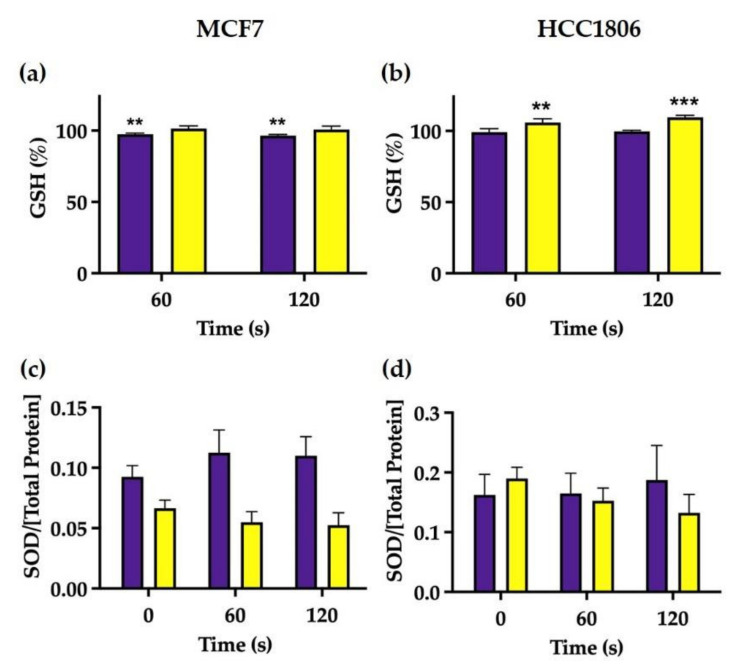
Intracellular GSH expression (**a**,**b**) and SOD activity (**c**,**d**) in MCF7 (**a**,**c**) and HCC1806 (**b**,**d**) cells were measured 2 and 24 h after treatment. GSH results are presented normalized to the value 100% corresponding to control cell cultures not exposed to cold atmospheric plasma (CAP). Data represent the mean ± SE of at least two independent experiments on both cell lines. Statistically significant differences are shown with ** *p* < 0.01 and *** *p* < 0.001.

As seen in Figure 4a,b, GSH levels were not different between CAP-treated cells and controls in the first 24 h following CAP exposure. In addition, during the same time window, we could not observe significant differences in the activity of SOD in both MCF7- and HHCC1806-treated cells, as depicted in Figure 4c,d.

### 2.4. CAP Treatment May Induce Cell Cycle Arrest in G2/M Phase and Reduce Long-Term Survival

**Figure 5:**
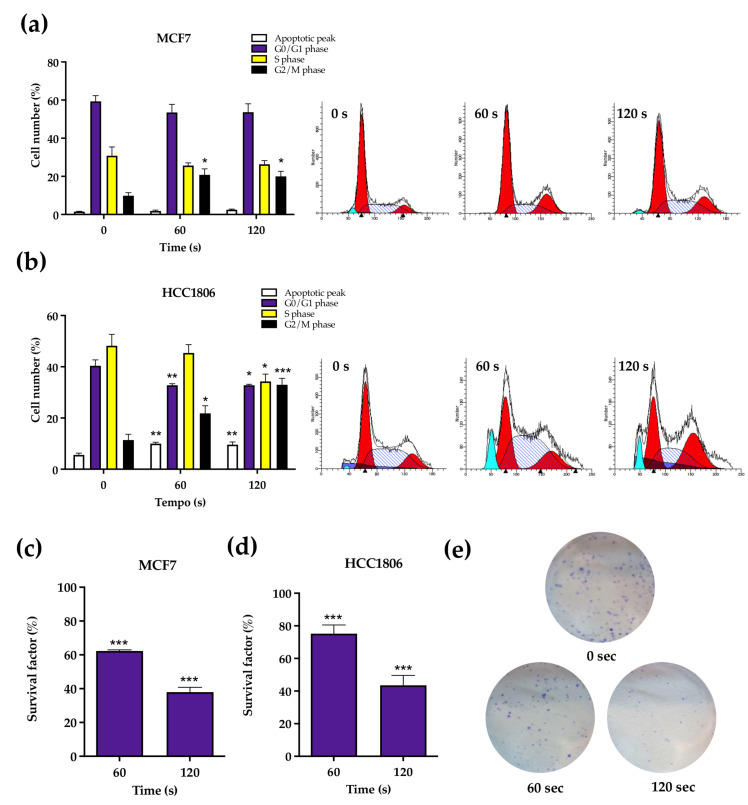
Cell cycle (**a**,**b**) and survival factor (**c**,**d**) were assessed in MCF7 (**a**,**c**) and HCC1806 (**b**,**d**) cell lines after cold atmospheric plasma (CAP) exposure by flow cytometry and clonogenic assay, respectively. Results of cell cycle express the percentage of cells in apoptotic peak, G0/G1 phase, S phase and G2/M phase and represent the mean ± SE of two independent experiments. Survival factor is expressed as the mean ± SE of three independent assays normalized to the value 100% corresponding to control cell cultures not exposed to CAP. Representative images of the congenic assay plates from MCF7 are presents (**e**). Statistically significant differences are shown with * *p* < 0.05, ** *p* < 0.01 and *** *p* < 0.001.

The cell cycle of MCF7 cells was generally little affected by plasma exposure at 60 and 120 s. However, a significant increase was observed in the cell population numbers in G2/M phase compared to the control, from 9.83 ± 1.62% to 20.83 ± 3.12% (*p* = 0.017) after 60 s of exposure and to 20.00 ± 2.70% (*p* = 0.028) after 120 s of exposure. Furthermore, HCC1806 cells significantly increased the cells in apoptotic peak, from 5.60 ± 0.68% to 10.00 ± 0.55% (*p* = 0.036) after 60 s of CAP therapy and to 9.60 ± 1.03% (*p* = 0.004) after 120 s. Similarly, there was a significant decrease in the cells in G0/G1 phase in both periods of CAP exposure, from 40.40 ± 2.29% to 32.80 ± 0.58% (*p* = 0.0095) after 60 s and to 32.80 ± 0.37% (*p* = 0.018) after 120 s. The S/phase showed a decrease from 48.20 ± 4.47% to 34.20 ± 2.84% (*p* = 0.034) after 120 s of CAP exposure. The G2/M phase showed a significant increase in the number of cells after 60 and 120 s, from 11.40 ± 2.25% to 21.80 ± 2.97% (*p* = 0.015) and 33.0 ± 2.49% (*p* = 0.0001), respectively. All results were expressed in Figure 5a,b.

As seen in Figure 5c,d, survival factor decreased in both cell lines in a dose-dependent manner according to CAP time exposure. Regarding the control, MCF7 cells’ survival factor decreased to 62.27 ± 0.68% (*p* = 0.013) and 37.92 ± 2.87% (*p* < 0.001) after 60 and 120 s of treatment, respectively. Similarly, HCC1806 cells’ survival factor decreased significantly to 75.20 ± 5.36% (*p* = 0.006) following 60 s of exposure and to 43.55 ± 6.11% (*p* < 0.001) following 120 s of exposure.

## 3. Discussion

Over the past decade, CAP studies have emerged as a new therapeutic approach in various types of cancer, highlighting its anti-proliferative effect [21,22,23], and several authors also describe the selective potential of therapy for tumour cells, preserving adjacent phenotypically normal cells [24,25].

Our previous results have studied the effect of CAP exposure in metabolic activity and viability of MCF7 and HCC1806 cells [19]. By comparing the metabolic activity of several cell lines after plasma exposure, we observed that longer CAP exposures were required to reduce metabolic activity in breast cancer cells than most of the cells tested [19]. However, the effect of plasma treatment in the cells’ viability differed between triple-negative (MCF7) and hormonal-receptor-positive (HCC1806) breast cancer cell lines. While only longer exposures decreased the viability of HCC1806 cells, parallel to the previous results, a short plasma exposure caused a marked and disproportional reduction in the MCF7 cell number. Thus, MCF7 cells were assumed to be stressed. Since plasma reduced the proliferation rate but failed to produce an equivalent reduction in metabolic activity under these conditions, stressed MCF7 cells displayed an increased metabolic rate per cell. The selection of a highly metabolic subpopulation of MCF7 cells or adaptation of remaining cells with increased mitochondrial biogenesis was hypothesized. Whichever the mechanism explaining the increase in metabolic index, plasma exposure to longer duration abolished this response. This work further explores the possible mechanisms of action of new plasma-based therapies in these breast cancer cell lines [19].

We have selected referred time exposures because we previously documented this differential response at 60 and 120 s. The presented results confirm the previously described reduction in cell viability after CAP exposure [19]. Firstly, we analysed the type of cell death in each cell line. For both cell lines and therapy durations, the proportion of viable cells in both cell lines were reduced. However, while apoptosis was the main type of death in MCF7 cells, a combination of apoptosis and necrosis was seen in HCC1806 cells. The evaluation of mitochondrial potential showed that at least some portion of treated cells lost their normal mitochondrial inner membrane electrical potential, a feature of apoptosis, consistent with the increased proportion of this type of death in both cell lines. An abrupt modification of MMP, as seen particularly in HCC1806, can drop adenosine triphosphate (ATP) concentration to levels insufficient for cell survival, not allowing the apoptotic process to end [26] and leading to necrosis. Apoptosis in response to plasma was also supported by the documentation of the overexpression of proapoptotic protein BAX when compared to antiapoptotic Bcl-2 protein and a reduction in procaspase-3 expression, a surrogate marker of its cleavage and conversion into the executioner caspase-3, the main effector of apoptosis [27]. Interestingly, the baseline BAX/Bcl2 ratio was intrinsically lower in MCF7 cells and did not significantly change when these cells were exposed to the shortest exposure. 

Since the main hypothesized mechanism of plasma therapy involves reactive oxidative and nitroxidative species (RONS), we determined the intracellular concentrations of these species shortly (2 h) and 24 h after exposure. Our previous works [19,20] suggests that H_2_O_2_ and NO_2_ are generated inside the plasma plume or in the interface between plasma and liquid and then solubilized in culture media. Then, we assume that RONS concentrations inside cells can be increased by (1) scavenging reactive species from media briefly after plasma irradiation or (2) mitochondria release by a mitochondrial-induced mitochondrial release mechanism later [28]. Conversely, intracellular RONS concentrations can be decreased by (1) consumption through conversion in other species shortly after treatment or (2) resetting the equilibrium following alterations between production and removal rate of RONS.

We detected distinct patterns in nitroxidative states between cell lines. Shortly after irradiation, HCC1806′s intracellular NO concentrations increased and O_2_^−^ concentrations decreased, while the inverse pattern occurred 24 h after treatment coupled with an increase in GSH levels. Perhaps the only noticeable difference between HCC1806 cells irradiated during 60 and 120 s was peroxide concentration, which was only slightly decreased in cells exposed longer to plasma. Differently, shortly after irradiation, MCF7 intracellular peroxide concentration and glutathione levels were equally decreased in cells exposed to 60 and 120 s. However, 24 h later, MCF7 cells behaved in different ways, with increased peroxide concentration seen in cells exposed to 60 s and decreased NO and O_2_^−^ concentrations in those exposed to the highest treatment duration. 

Several RONS have been identified in cell media exposed to plasma, but most are short-lived. NO_2_^−^ and H_2_O_2_ are long-lived species and are thought to be the major effectors of plasma effects [29]. We previously determined peroxide concentrations in irradiated cell media of about 1–4 mM with a single exposure of 120 s [20]. However, intracellular H_2_O_2_ concentrations were not increased, contrary to what we have seen in previous works with retinoblastoma cells [20]. These paradoxical results were also reported by other authors, reinforcing the idea that some cancer cells are more resistant to oxidative species, thus suggesting a highly effective antioxidative system [30]. We therefore measured GSH levels and did find a decrease in MCF7 cells paralleling a decrease in intracellular peroxides, suggesting a mutual consumption reaction. HCC1806 cells increased GSH levels 24 h after plasma exposition, compatible with a reactive production of these proteins capable of detoxifying peroxides. However, these variations in GSH levels, albeit statistically significant, were marginal and directed our attention to other antioxidative defences that could better explain the antioxidative capacity of these breast cancer cell lines. Based on previous reports [30], we tested the role of mitochondrial cytochrome c oxidase. Biochemical inhibition of cytochrome c oxidase activity was performed with NaN_3_ in a non-cytotoxic concentration. Incubation with NaN_3_ and while irradiating with plasma was able to achieve an accentuated reduction in metabolic activity following a 60 s exposure. This was particularly relevant since plasma per se did not achieve a significant reduction in metabolic activity of breast cancer cells [19]. Thus, plasma resistance was reduced to levels comparable to cell lines regarded as susceptible. While NaN_3_ may also quench singlet oxygen derived from photodynamic reactions between endogenous photosensitizers and UV light photons emitted by plasma [20,31], this mechanism would not increase the enhanced cell killing capacity of plasma therapy as seen in our data since it would abrogate a generation of oxidative damage. An interesting observation was an increase in peroxides 24 h after plasma exposure in MCF7 cells and following restauration of GSH levels, which may represent a new balance in the redox state of this cells, which can be advantageous in highly metabolically active cells, since oxidized cytochrome c oxidase can bind hydrogen peroxide and use it as an electron acceptor in mitochondrial electronic chain [32,33]. 

While there is NO in the plasma plume, it is not soluble in medium, and therefore it is not scavenged by cells. NO is thought to be produced inside cells from the conversion of NO_2_ produced in the plasma plume and solubilized in medium. Thus, our results suggest that HCC1806 cells scavenge and metabolize NO_2_, pointing out that these cells, but not MCF7 cells, have NO_2_^−^ reductase activity, which can be used to enhance plasma toxicity. Superoxide anions seem to inversely correlate with NO, which suggests that it might be consumed in a reaction with NO to form peroxynitrite (ONOO^−^). Note that NO is the only molecule produced by biological systems in high enough concentrations to out-compete superoxide dismutase for superoxide [34], possibly explaining why SOD levels do not change in HCC1806 cells. 

These results combined suggest that both cell lines are relatively resistant to high concentrations of peroxides produced inside plasma due to the peroxidase activity of cytochrome c oxidase and can be made susceptible by addition of NaN_3_. Furthermore, optimal midrange peroxide concentration can help to boost the metabolic index of viable MCF7 as an electron acceptor in the mitochondrial electron transport chain. At last, the nitrate reductase activity of HCC1806 cells may partially explain the effects of CAP in this cell line.

We next explored the cell cycle distribution of both cell lines, which revealed subtle differences between the two. Both cell lines display an increase in G2/M, which can be explained by an arrest in this phase following the activation of cell cycle checkpoints after the detection of damages in DNA replication possibly induced by RONS. 

At last, clonogenic assay results, the gold standard for long time survival, expanded the observed cytotoxic results of atmospheric plasma to the long term. These results include MCF7 cells exposed to CAP for 60 s, thus suggesting that their enhanced metabolic index does not translate into enhanced survival. 

## 4. Materials and Methods

### 4.1. Cell Culture

In this study, we used two adherent human cancer cell lines representative of two distinct subtypes of BC, namely, the hormonal-receptor-positive breast cancer cell line MCF7) and triple-negative breast cancer cell line HCC1806. These cell lines were purchased from American Type Culture Collection (ATCC, Manassas, VA, USA) and were cultured under standard cell culture conditions (37 °C, 5% CO_2_). MCF7 cells were cultured with Dulbecco’s Modified Eagle Medium (Sigma Aldrich, St. Louis, MO, USA) medium, and HCC1806 cells were maintained in Roswell Park Memorial Institute (Sigma Aldrich, St. Louis, MO, USA) medium, according to suppliers’ recommendations. Media were supplemented with 5% foetal bovine serum (Sigma Aldrich, St. Louis, MO, USA) and 1% of penicillin-streptomycin solution (10,000 U/mL penicillin, 10 mg/mL streptomycin, and 25 μg/mL amphotericin B; Sigma Aldrich, St. Louis, MO, USA).

### 4.2. CAP Treatment

An electronic device capable generating CAP into cell culture plates was developed at the Institute of Biophysics, Faculty of Medicine, University of Coimbra, as described in detail elsewhere [19]. Briefly, this device generates high voltage (4 kV) pulses with a frequency of 1 KHz through a sterilized needle with 0.9 mm of radius and 40mm of length (Microlance 3, Becton Dickinson, Franklin Lakes, NJ, USA). When charged, the needle works as an open-air single electrode CAP jet. An electrically grounded needle was submerged in the culture media. In this design, the cultures act both as target and grounded electrode, enabling plasma generation. The high voltage needle was placed 2mm above the surface of the cell culture’s medium. Cells were platted in 24-well plates (Sarstedt, Nümbrecht, Germany) at a density of 500,000 cells/mL in a volume of 500 μL per well and submitted to CAP treatment for short periods of time: 60 and 120 s. Further assays were performed 2 or 24 h after the exposure.

### 4.3. Cell Viability

Twenty-four hours after CAP treatment, samples with 10^6^ cells were centrifuged for 5 min at 1300× *g*. The pellet was suspended in 100 µL of binding buffer, constituted by 0.01M Hepes (Sigma Aldrich, St. Louis, MO, USA), 0.14M NaCl (Sigma Aldrich, St. Louis, MO, USA) and 0.2 mM CaCl_2_ (Sigma Aldrich, St. Louis, MO, USA). Then, cells were labelled with annexin-V-fluorescein isothiocyanate (AnV-FITC) and propidium iodide (IP) as recommended by the kit supplier (Immunotech, Marseille, France). Samples were analysed in a four-color flow cytometer FACSCalibur (Becton Dickinson, New Jersey, NY, USA). 

### 4.4. Mitochondrial Membrane Potential

Twenty-four hours after CAP exposure, 10^6^ cells were centrifuged for 5 min at 1300× *g*, and the samples were incubated with 40 µL of fluorescent probe JC-1 (5,5′,6,6′-tetrachloro-1,1′,3,3′-tetraethylbenzimidazolocarbocyanine iodide; Invitrogen^®^, Waltham, MA, USA) for 15 min at 37 °C in the dark. After washing, cells were evaluated in the flow cytometer. For monomers, the fluorescence was determined at 485/20 nm excitation and 528/20 nm emission wavelengths, being the aggregates fluorescence determined at 530/25.5 nm excitation and 590/35 nm emission wavelengths. The results are expressed as the variation of the monomers/aggregates (M/A) ratio regarding each experimental control.

### 4.5. BAX and BCL2 Proteins Expression

Twenty-four hours after CAP treatment, samples with 10^6^ cells were centrifuged for 5 min at 1300× *g* and cells were fixed and permeabilized with the Intracell Kit (Immunostep Biotech, Salamanca, Spain), as recommended by the supplier. Samples were incubated with 0.6 µg of anti-BAX-PE antibody (Santa Cruz Biotechnology, Santa Cruz, CA, USA) and 0.6 µg of anti-BCL2-FITC (Santa Cruz Biotechnology, Santa Cruz, CA, USA) for 15 min at room temperature. Detection was performed by flow cytometry.

### 4.6. Caspase 3

Protein extracts were prepared using an appropriate lysing buffer and the protein concentration was determined by BCA method (Pierce™ BCA Protein Assay Kit, ThermoFisher Scientific, Porto Salvo, Portugal). Denaturated protein samples were run on electrophoresis gel during 15 min at 80 V and 1 h 15 min at 150 V, followed by the electrotransfer to PVDF membranes (Immun-Blot^®^ PVDF Membrane, Bio-Rad, Amadora, Portugal). Caspase 3 was detected with a primary monoclonal antibody anti-caspase 3 (Santa Cruz Biotechnology, Santa Cruz, CA, USA) and succeeding a suitable secondary antibody anti-mouse. Finally, membranes were incubated with ECF substrate (GE Healthcare, Chicago, IL, USA) and revealed using a fluorescence reader Typhoon FLA 9000 (GE Healthcare, Chicago, IL, USA).

### 4.7. Reactive Oxygen and Nitrogen Species (RONS)

Two and 24 h after CAP therapy, 10^6^ cells were centrifuged for 5 min at 1300× *g* and washed with phosphate saline buffer (PBS), constituted of 137 mM NaCl (Sigma Aldrich, St. Louis, MO, USA), 2.7 mM KCl (Sigma Aldrich, St. Louis, MO, USA), 10 mM Na_2_HPO_4_.2H_2_O (Sigma Aldrich, St. Louis, MO, USA), 2.0 mM KH_2_PO_4_ (Sigma Aldrich, St. Louis, MO, USA). The tests were performed by fluorescence in a multiwell plate spectrophotometer (Synergy HT, Winooski, VT, USA).

#### 4.7.1. Intracellular peroxides

Cells were incubated with 5 μM of 2′,7′-dichlorodihydrofluorescein diacetate (Invitrogen^®^, Waltham, MA, USA) probe for 45 min in the dark at 37 °C. After washing, samples were analysed with the excitation and emission wavelengths of 485 and 520 nm, respectively.

#### 4.7.2. Intracellular Superoxide Anion

Cells were incubated with 2 μM of dihydroethidium (Sigma Aldrich, St. Louis, MO, USA) probe for 15 min in the dark at 37 °C. After washing, detection was performed using the excitation and emission wavelengths of 500 and 645 nm, respectively.

#### 4.7.3. Intracellular Nitric Oxide

Cultures were incubated with 1 μM of 4-amino-5-methylamino-2′,7′-difluorofluorescein diacetate (Invitrogen^®^, Waltham, MA, USA) for 1 h in the dark at 37 °C. After washing, reading was performed with excitation and emission wavelengths of 495 and 515 nm, respectively.

### 4.8. Cytochrome c Oxidase Inhibition

Cells were incubated with 1 mM sodium azide (Sigma Aldrich, St. Louis, MO, USA) two hours prior CAP exposure. After CAP treatment, cells were incubated for 30 min and cell media renewed. Twenty-four hours after CAP exposure, cell cultures were washed with PBS, and 0.5 mg/mL of MTT (Sigma Aldrich, St. Louis, MO, USA) was added in each well. Cells were incubated at 37 °C, at least 4 h in dark, followed by gentle agitation with an isopropanol solution (Sigma Aldrich, St. Louis, MO, USA). Absorbance was read at wavelengths of 570 and 620 nm (Sigma Aldrich, St. Louis, MO, USA). It is important to note that previous studies were carried out in order to assess the non-toxic concentration of sodium azide for each cell line. A concentration of 1 mM of sodium azide solution was selected based on results displaying preserved metabolic activity of cells exposed to this solution (96.27 ± 9.06% in HCC1806 cells and 81.54 ± 4.24% in hormone-dependent HCC1806 cells).

### 4.9. Anti-Oxidative Defences

The evaluation of anti-oxidative defences was performed 2 and 24 h after CAP treatment. Samples with 10^6^ cells were centrifuged for 5 min at 1300 × *g* and washed with PBS.

#### 4.9.1. GSH

Cells were incubated with 10 μM of mercury orange (Sigma Aldrich, St. Louis, MO, USA) for 15 min in the dark at 37 °C. Samples were read with excitation and emission wavelengths of 485 and 590 nm, respectively, in the multi-well plate spectrophotometer.

#### 4.9.2. SOD

Cell lyses was promoted with RIPA buffer, constituted of 25 mM Tris-HCl (Sigma Aldrich, St. Louis, MO, USA), 150 mM NaCl (Sigma Aldrich, St. Louis, MO, USA), 1% NP-40 (Sigma Aldrich, St. Louis, MO, USA), 1% sodium deoxycholate (Sigma Aldrich, St. Louis, MO, USA), 0.1% SDS (Sigma Aldrich, St. Louis, MO, USA) was added to cells. Cells were sonicated for 10 cycles of 3 s, each with an amplitude of 40%, centrifuged for 18 min at 14,000× *g* and the supernatant stored at −80 °C. Protein concentration was determined as already described. SOD activity was measured using the SOD Assay Kit-WST (Sigma Aldrich, St. Louis, MO, USA) according to suppliers’ protocol.

### 4.10. Cell Cycle

Twenty-four hours after CAP exposure, samples with 10^6^ cells were centrifuged for 5 min at 1300× *g* and fixed in 70% ethanol (Sigma Aldrich, St. Louis, MO, USA) for 30 min at 4 °C. After washing, a propidium iodide PI/RNase solution (Immunostep, Salamanca, Spain) was added and incubated during 15 min. Samples were evaluated in the flow cytometer.

### 4.11. Clonogenic Assay

After CAP treatment, cells were incubated for 2 h. Then, 2000 cells were platted in 6-well plates (Sarstedt, Nümbrecht, Germany) with 3 mL of medium. After 15 days in MCF7 cell line and after 18 days in HCC1806 cell line, cells were fixed with methanol and wells were stained with 2 mL of crystal violet dye (Sigma Aldrich, St. Louis, MO, USA). Individual colonies were counted for each well and survival factor calculated according to Equation (1):

(1) $$\mathrm{Survival}\;\mathrm{factor}\;\left(\%\right)=\frac{\frac{\mathrm{Colonies}\;\mathrm{counted}\;\mathrm{in}\;\mathrm{CAP}\;\mathrm{treated}\;\mathrm{wells}}{\mathrm{Cells}\;\mathrm{seeded}\;\mathrm{in}\;\mathrm{CAP}\;\mathrm{treated}\;\mathrm{wells}}\;\;}{\frac{\mathrm{Colonies}\;\mathrm{counted}\;\mathrm{in}\;\mathrm{control}\;\mathrm{wells}}{\mathrm{Cells}\;\mathrm{seeded}\;\mathrm{control}\;\mathrm{wells}}}$$

### 4.12. Statistical Analysis

Statistical analysis was performed using Prism 9 (San Diego, CA, USA). The normality of the distribution of quantitative variables was assessed according to the Shapiro–Wilk test. Parametric tests were used in case of a normal distribution, and nonparametric tests were used otherwise. The comparison of results with a hypothetical value (experiments where control was normalized to 1 or 100) using the one sample t test if the sample assumed a normal distribution and the Wilcoxon test if the last condition was not met. The comparison of quantitative variables between more than two groups was obtained with the ANOVA 1 Fixed Factor test (parametric test) or the Kruskal–Wallis test (non-parametric test). Multiple comparisons were corrected with Holm–Šídák’s or Dunn’s multiple comparisons test, as applicable. A significance value of 0.05 was considered for all comparisons. 

## 5. Conclusions

The results presented throughout this study highlight the therapeutic potential of cold atmospheric plasma and contributed to clarifying possible mechanisms of this treatment in breast cancer cells. Different effects of plasma therapy between MCF7 and HCC1806 cells seen previously in our work were confirmed. Apoptosis seems to be the most prevalent type of cell death after CAP treatment, while HCC1806 cell line also showed a significant proportion of cells undergoing necrosis. Cell cycle arrest may occur in G2/M after cumulative defects in DNA replication. The evaluation of RONS suggest that breast cancer cells have highly active antioxidative systems, which can be overcome by selective inhibition with sodium azide. Furthermore, elevated nitric oxide concentrations were only seen in HCC1806, suggesting intrinsically higher nitrite reductase activity. Moreover, decreased long-term survival was documented and independent of the histological type of breast cancer cell line. In the future, based on these results, the assessment of tumour growth in vivo and its side effects in an animal model will be an important next study for the advancement in a clinical approach. 

## Data Availability

Data are contained within the article.
